# Turn down of acute aortic syndrome cases during COVID‐19: Results from UK multicentre studies

**DOI:** 10.1111/jocs.15187

**Published:** 2020-11-10

**Authors:** Marius Roman, Amer Harky, Andrew Brazier, Kelvin Lim, George Gradinariu, Aung Oo, Giovanni Mariscalco, Ana Lopez‐Marco

**Affiliations:** ^1^ Department of Cardiothoracic Surgery Glenfield Hospital Leicester UK; ^2^ Department of Cardiothoracic Surgery Liverpool Heart and Chest Hospital Liverpool UK; ^3^ Department of Integrative Biology, Faculty of Life Sciences University of Liverpool Liverpool UK; ^4^ Liverpool Heart and Chest Hospital, Liverpool Centre for Cardiovascular Science, University of Liverpool Liverpool UK; ^5^ Department of Cardiothoracic Surgery University Hospital Coventry Coventry UK; ^6^ Department of Cardiothoracic Surgery The Royal Infirmary of Edinburgh Edinburgh UK; ^7^ Department of Cardiothoracic Surgery Aberdeen Royal Infirmary Aberdeen UK; ^8^ Department of Cardiothoracic Surgery St Bartholomew's Hospital London UK

**Keywords:** acute aortic syndrome, conservative treatment, COVID‐19 pandemic, surgery

## Abstract

**Objective:**

The coronavirus disease 2019 (COVID‐19) pandemic has restructured the healthcare systems, prioritizing resources to treat COVID‐19 patients. The aim of this study was to establish if patients affected by acute aortic syndrome (AAS) had unrestricted access to emergency treatment and evaluate outcome of these patients during the peak of the pandemic.

**Methods:**

This is a retrospective analysis of prospectively collected data between March and June 2020 from 19 participating cardiac surgery centers in the United Kingdom.

**Results:**

Among 95 patients who presented with an AAS in the participating centers; 85 (89%) underwent surgery, 7 (7%) were turned down for surgery because of their profile of comorbidities, and 3 (3%) died on transfer. Among the patients treated conservatively, three of them (43%) were alive at 30 days. We observed no significant restriction in access to treatment for AAS during the early months of the pandemic.

**Conclusion:**

Services for life‐threatening aortic surgery patients were maintained during the COVID‐19 period through patient selection and timing of surgery. The rate of surgical turn‐down was comparable to published figures despite the challenges faced during the COVID‐19 pandemic.

## 1 INTRODUCTION

Severe acute respiratory syndrome Coronavirus 2, the virus that causes coronavirus disease 2019 (COVID‐19), was first described as a cluster of cases of pneumonia in the city of Wuhan ‐ China on December 31, 2019. The number of cases exponentially increased and spread rapidly to other geographical locations, reaching global pandemic status on March 11, 2020, which has led to a collective global effort to tackle this pandemic and accelerating research in this field.^1^

An extensive restructuring of the healthcare services has taken place since the declaration of the COVID‐19 pandemic due to the need for reallocation of the hospital and intensive care resources to patients affected by COVID‐19, as well as to protect healthy individuals from unnecessary exposure in hospital environments. In the United Kingdom, the majority of the units were prompted to put all elective surgery on hold during March and April 2020, leading to a restructuring in the provision of aortovascular services during the pandemic to protect access to essential emergency treatment.^2^

Acute aortic syndromes (AAS) represent a surgical emergency, with a devastating natural history carrying high mortality (1%–2% each hour) if not treated immediately.^3^, ^4^ The aim of this study was to establish if AAS patients had unrestricted access to emergency treatment and the outcomes of these patients during the peak of the pandemic.

## 2 METHODS

This is a retrospective analysis of prospectively collected data from a multicentre study with 19 participating aortic centers from the United Kingdom (66% of the aortic units in the country, including the largest specialized aortic centers and covering most of the geographical areas), designed to assess the impact of the COVID‐19 pandemic in the delivery of services for aortovascular disease in the United Kingdom between March and June 2020.

The other 15 aortic units declined to participate in the study for different reasons including the inability to provide emergency surgery cover during the COVID‐19, insufficient resources to collect the data, and/or individual preferences.

We identified patients who were admitted to specialized aortic units with the diagnosis of AAS and turned down for surgery and clinical outcomes for these patients when compared to the outcomes of the surgical cohort in the same period.

## 3 RESULTS

Between March and June 2020, 95 patients were admitted with a diagnosis of AAS at the participating centers. Seven patients (7.3%) were turned down for surgery. The mean age was 77.4 years (74–81) and 57% were females. Six patients presented with an acute aortic dissection (five DeBakey I and one DeBakey II) and one with an acute on chronic DeBakey III aortic dissection. Five patients (71%) had undergone prior cardiac surgery.

The rationale for the surgical turn down was based on the following reasons: clinical complexity, frailty or multi‐comorbidities, and history of previous cardiac surgery. Only one patient was diagnosed with COVID‐19 disease based on radiological finidings of ground‐glass opacities on the computed tomography scan and therefore the decision of not offering surgery, as COVID‐19 considered as an additional increase in his perioperative risk profile.

The mean EuroScore II was 18.9 (4.8–37.0). Four patients (57%) died as a result of aortic dissection (aortic rupture [75%] and malperfusion [25%]). Three patients (43% of turn‐down patients) survived with conservative management and were discharged home (Table 1).

**Table 1:** Demographics, diagnosis, and prior medical history for patients turned down for surgery

Patient	Sex	Age	Diagnosis	Medical history	Covid status	EuroScore	Reason for turn down	Outcome
1	M	78	Acute DeBakey I	MI	Negative	18.2	Comorbidities	Alive
				TIA				
				COPD				
				Previous AVR + CABG				
2	F	79	Acute DeBakey I	Dialysis	Negative	24.7	Comorbidities	Died
				Previous AVR + CABG				
3	F	74	Acute DeBakey I	Smoker	Unknown	26.1	Comorbidities	Died
				Previous aortic root replacement				
4	F	74	Acute DeBakey I	Hypertension	CT changes suspicious for COVID‐19	10.8	COVID status	Died
				Ex‐smoker				
5	F	75	Acute DeBakey II	Hypertension	Negative	4.8	Comorbidities	Alive
				CKD				
6	M	81	Acute DeBakey I	Hypertension	Negative	10.8	Comorbidities	Alive
				Previous AVR + CABG				
7	M	81	Acute on chronic DeBakey III	Marfan syndrome	Unknown	37.0	Comorbidities	Died
				Previous aortic dissection repair and infrarenal AAA repair				

Abbreviations: AA, ascending aorta; AAA, abdominal aortic aneurysm; AVR, aortic valve replacement; CABG, coronary artery bypass grafting; CKD, chronic kidney disease; COPD, chronic obstructive pulmonary disease; COVID‐19, coronavirus disease 2019; CT, computed tomography; F, female; M, male; MI, myocardial infarction; TIA, transient ischemic attack.

These patients presented in four different aortic centers, two of them leading aortic centers in volume and expertise. The two leading centers had a turn‐down rate of 10% and 12%, while the other two centers turned down 50% and 67% of the AAS referred to them.

The rest of the participating centers did not turn down for surgery any of the referred patients, but no cases with previous cardiac or aortic surgery were admitted to these units during this period.

Eighty‐five patients (89.4%) with AAS underwent surgery. The mean age was 62 years (29–83 years) and 32% were females (*n* = 27). The mean EuroScore was 9.6 (1.8–40.8; Table 2). The surgical procedures performed were aortic valve and ascending aorta replacement (*n* = 6), aortic root replacement (*n* = 32), ascending aorta and hemiarch replacement (*n* = 27), total arch replacement (*n* = 18), frozen elephant trunk repair of descending thoracic aorta (*n* = 8), descending thoracic aorta replacement (*n* = 4), and thoracic endovascular aortic repair (*n* = 2). In‐hospital mortality in this cohort was 25% (*n* = 21), with a 9.5% rate of intraoperative deaths (*n* = 8).

**Table 2:** Demographics, cardiovascular risk factors, and anatomy of the acute aortic syndrome at presentation

	Operated	Not operated
Age	62 (29–83)	77.4 (74–81)
Female sex	26 (32%)	(57%)
COPD	7 (8%)	1 (14%)
Prior stroke	2 (2%)	1 (14%)
Poor LV function	3 (3%)	0
Prior surgery	12 (14%)	5 (71%)
EuroScore	9.6 (1.8–40.8)	18.9 (4.8–37.0)
DeBakey I	71 (83%)	5 (71%)
DeBakey II	7 (8%)	1 (14%)
DeBakey III	10 (12%)	1 (14%)

Abbreviations: COPD, chronic obstructive pulmonary disease; LV, left ventricle.

Three other patients (3.1%) with an acute DeBakey I aortic dissection were accepted for emergency surgery but died of aortic rupture and cardiac tamponade while waiting for transfer to the specialized aortic unit or during the anesthetic induction. The mean age for this group was 61.3 years (45–75 years) and the mean EuroScore II was 12.6 (4.8–23).

Surgical activity for AAS was overall maintained during the early months of the COVID‐19 pandemic in the UK among the participating centers (88 cases during the study period vs. 80 cases operated during the same period last year). The majority of centers (58%) operated an equivalent number of patients (±1) during the same period last year, while five centers experienced a reduced activity of 2–4 cases when compared to prepandemic figures. Only one of the centers, exceeded its aortic emergency activity during the pandemic by 15 patients when compared to the previous year due to being designated as a reference center providing regional cover for emergency cardiac and aortic surgery.

The surgical outcomes during the pandemic period were benchmarked with national data for aortic dissection survival provided by the UK AS group and were deemed comparable to prepandemic figures.

## 4 DISCUSSION

The restructuring of healthcare systems that have taken place during the COVID‐19 pandemic to allocate intensive care resources primary to COVID‐19 patients, has put all the elective surgery on hold during the early part of the pandemic in the United Kingdom.^2^

However, emergency conditions like AAS, have been continuously referred to the specialized cardiac and aortic centers for assessment and management.

The natural progression of AAS disease carries an increasing mortality,^3^, ^4^, ^5^ leading to logistical challenges in offering adequate medical care and treatment due to possible delays in determining the COVID‐19 status for these patients. In our cohort, we observed a personalized practice for each of these patients, weighing the benefits and risks of developing COVID‐19 in the immediate postoperative period versus the risk of spontaneous rupture and time‐dependent increasing mortality while waiting for the screening results.

The majority of patients who presented with an AAS received emergency surgical treatment and the outcomes were consistent with the prepandemic period (25% mortality)^4^, ^6^ despite the challenges faced during this crisis: generalized reluctance to attend A&E services, delays in interhospital transfers due to increased workload of the ambulance services, delays in anesthetic, and surgical times due to the universal COVID‐19‐related precautions.

The majority of the patients who were turned down for surgery presented to the hospital during the first month of the pandemic in the United Kingdom. Once the logistical challenges were resolved, the surgical turn‐down rate significantly reduced in the last months of the pandemic.

Explanations for the turn down for surgery during the first month were related to the uncertainty and the ability to quantify the additional perioperative risks related to COVID‐19 infections, in the context of patients with multiple comorbidities and undergoing a high‐risk procedure. There were, however, no surgical turn downs directly related to the relocation of resources due to the COVID‐19 pandemic (i.e., shortage of intensive care unit beds or healthcare professionals due to relocation treat respiratory patients).

The international registry of acute aortic dissection reported surgical correction in only 86% of their cohort among the total of 2952 patients presented with acute type A aortic dissection^6^; it is important to note that our turn‐down rate of 7.3% during COVID‐19 is reflecting an acceptable figure considering the challenging times of the COVID‐19 pandemic. We predicted a higher turn‐down rate during the pandemic considering the challenges that the pandemic has posed as highlighted above.

It is well known that surgery is the mainstay of treatment for DeBakey I and II and complicated DeBakey III AAS, and the literature reports a high mortality for those who are treated conservatively.^3^, ^4^, ^5^, ^6^, ^7^

Forty‐three percent of the patients turned down for surgery survived the AAS episode with conservative management allowing for further planning of treatment options for their disease.

Previous cardiac and/or aortic surgery was one of the main reasons for turning patients down for surgery as these patients pose a management challenge in the setting of an AAS. The presence of mediastinal adhesions and scarring theoretically prevent them from an aortic rupture and cardiac tamponade. Moreover, a redo sternotomy and exposure of the aortic tissues during out‐of‐hours and without a specialized on‐call aortic service can be challenging.

These complex patients presenting with AAS in the context of previous cardiac surgery might be better served by delaying the emergency treatment to allow more comprehensive planning for the definitive treatment by a specialized complex aortic team. In the highest volume specialised aortic centers in the United Kingdom, the approach to patients with previous surgery who present with AAS is to admit then to an intensive care setting to monitor and treat the blood pressure while requesting additional diagnostic tests (e.g., a patient with previous coronary surgery required investigation of graft patency to plan the surgical strategy and myocardial protection). These cases are often discussed in an ad‐hoc multidisciplinary team (MDT) meeting. Other AAS, aside from acute DeBakey I and II aortic dissections, are routinely discussed in the aortovascular MDTs, which are run in different formats (e.g., virtual or face to face) and frequency (weekly or bi‐weekly) among the participating centers.

## 5 LIMITATIONS

There are several limitations associated with our study, including the small sample size in this subcohort or patients and the variation in practice among the participating centers.

## 6 CONCLUSIONS

There was no restricted access to treatment for AAS during the early months of the pandemic in the United Kingdom and the outcomes of patients surgically treated were consistent with prepandemic figures.

The surgical turn‐down rate for patients with AAS has been lower than expected despite the challenges the pandemic posed and not directly related to COVID‐19 status.

## CONFLICT OF INTERESTS

The authors declare that there are no conflicts of interest.
